# Origin of Polyploidy, Phylogenetic Relationships, and Biogeography of Botiid Fishes (Teleostei: Cypriniformes)

**DOI:** 10.3390/biology14050531

**Published:** 2025-05-11

**Authors:** Lei Yang, Richard L. Mayden, Gavin J. P. Naylor

**Affiliations:** 1 Florida Museum of Natural History, University of Florida, 1659 Museum Rd., Gainesville, FL 32611, USA; 2 Biology Department, Saint Louis University, 3507 Laclede Avenue, St. Louis, MO 63103, USA

**Keywords:** allopolyploidy, genome, loach, mitogenome, nuclear, timetree

## Abstract

Botiidae is a small family of freshwater fishes distributed across Southeast Asia, South Asia, and East Asia. It comprises two subfamilies: the diploid Leptobotiinae and the tetraploid Botiinae. Whether species in the Botiinae are autotetraploids or allotetraploids and how many polyploidization events occurred during the evolution of this subfamily remain open questions. The phylogenetic relationships and biogeography of the Botiidae also require further investigation. In the current study, we compared phylogenetic trees constructed using DNA sequences from the mitochondrial genome and five phased nuclear genes. We also performed whole genome sequencing for two tetraploid species: *Chromobotia macracanthus* and *Yasuhikotakia modesta*. Genome profiling of five botiine species suggests that they are likely of allotetraploid origin. Nuclear gene tree topologies indicate that the tetraploidization of the Botiinae occurred only once in the common ancestor of this subfamily. Although the possible maternal progenitor and paternal progenitor of the Botiinae cannot be determined, the subfamily Leptobotiinae can be excluded as a progenitor. The gene trees built in this study generally agree on the following sister group relationships: Leptobotiinae/Botiinae, *Leptobotia*/*Parabotia*, *Chromobotia*/*Botia*, *Yasuhikotakia*/*Syncrossus*, and *Sinibotia*/*Ambastaia*. Clades formed by the last two generic pairs are also sisters to each other. Timetree analyses and ancestral range reconstruction suggest that the family Botiidae might have originated in East Asia and Mainland Southeast Asia approximately 51 million years ago and later dispersed to South Asia and the islands of Southeast Asia.

## Simple Summary

In the present study, we found that fishes in the subfamily Botiinae are likely of allotetraploid origin, meaning they possess four sets of chromosomes derived from distinct parental species. This tetraploidization event appears to have occurred only once, in the common ancestor of the subfamily. Additionally, our results provide new insights into the phylogenetic relationships and biogeographical history of the family Botiidae.

## 1. Introduction

As an important evolutionary and ecological force, polyploidy (whole genome duplication) has received more and more attention in recent years (e.g., [1,2,3,4]). Compared to plants, much fewer polyploids have been found in fishes [5,6], and research on polyploid fishes has lagged behind that of polyploid plants in many aspects. Polyploids can be broadly categorized into two main types: autopolyploids and allopolyploids. Autopolyploids possess more than two sets of chromosomes derived from a single species, whereas allopolyploids carry more than two sets of chromosomes originating from different species, often through hybridization followed by genome doubling. For some known polyploid fish groups (e.g., *Tor*, *Probarbus*, *Spinibarbus*, and their allies), it has been unclear whether they are autopolyploids or allopolyploids until relatively recently [7,8]. For some other polyploid fish groups, such as the polyploid members of the family Botiidae, the situation remains unresolved.

Botiidae is a primary freshwater fish family distributed across Southeast Asia, South Asia, and East Asia. Some species, such as the Clown loach (*Chromobotia macracanthus*), are very popular in the aquarium trade. The family currently comprises around 65 valid species in two subfamilies and eight genera [9]. The subfamily Leptobotiinae includes two genera, *Leptobotia* and *Parabotia*, while the subfamily Botiinae consists of six genera, *Ambastaia*, *Botia*, *Chromobotia*, *Sinibotia*, *Syncrossus*, and *Yasuhikotakia*. Early studies on the karyotypes of botiid species (e.g., [10,11,12,13]) found that species in Leptobotiinae are diploids (2n = 50), whereas species in Botiinae are tetraploids (2n = c.100). However, none of these studies addressed whether fishes in Botiinae are autotetraploids or allotetraploids. Kaewmad et al. (2014) [14] explicitly stated that they are autotetraploids but provided no convincing evidence to support this claim, merely citing the aforementioned early studies (i.e., [10,11,12]). In contrast, the molecular phylogenetic study by Šlechtová et al. (2006) and the molecular cytogenetic work by Sember et al. (2018) both concluded that the underlying mechanism of polyploidization in Botiinae remains unclear [15,16]. More recently, Bitsikas et al. (2024) and Lv et al. (2024) published the genome assemblies for several botiine species (i.e., *Chromobotia macracanthus*, *Yasuhikotakia modesta*, *Ambastaia sidthimunki*, *Botia almorhae*, *B. kubotai*, and *Sinibotia reevesae*) [17,18]. However, they did not comment on whether these species are autopolyploids or allopolyploids.

Over the years, numerous methods have been developed to distinguish the two types of polyploids. Earlier studies relied on taxonomic investigation, chromosome counts, and cytological observation (e.g., [19]). In recent years, however, most studies have employed molecular cytogenetics, molecular genetics, or genomics approaches. A detailed review of many of these methods and their limitations is provided in [20]. In the current study, we applied a phylogeny-based genetic method and a k-mer-based genomic method to investigate whether botiines are autotetraploids or allotetraploids. Previous studies have shown that comparing phylogenetic trees built from mitochondrial genes and nuclear genes can help distinguish different types of polyploidization [8,21,22]. That is because mitochondrial genes are maternally inherited, whereas nuclear genes are biparentally inherited. Such analyses require separating the different nuclear gene copies in polyploid species and using them to construct gene trees. The second method utilized GenomeScope 2.0 [23], which distinguishes autotetraploids from allotetraploids based on the relative abundance of different nucleotide heterozygosity forms in k-mers generated from unassembled sequencing reads.

Resolving the phylogenetic relationships of a relatively small group like Botiidae may superficially seem straightforward. Indeed, several phylogenetic studies have already been conducted on the Botiidae using either mitochondrial genes alone (e.g., [15,24]) or both mitochondrial and nuclear genes (e.g., [16,25]). However, the use of nuclear genes in phylogenetic studies of polyploid species presents significant challenges. Every single-copy nuclear gene has two copies (four alleles) in tetraploids, like the subfamily Botiinae. These gene copies need to be sorted out properly, and only orthologous sequences can be used to resolve sister relationships among taxa. Unfortunately, previous studies using nuclear genes (e.g., [25]) did not separate the different gene copies and, thus, may be compromised by paralogy issues. In the present study, we attempted to separate the different gene copies of five single-copy nuclear genes: EGR2B (early growth response protein 2B gene), EGR3 (early growth response protein 3 gene), RAG1 (recombination activating gene 1, exon 3), RAG2 (recombination activating gene 2), and IRBP2 (interphotoreceptor retinoid-binding protein gene 2). We also reconstructed the phylogenetic relationships of botiids using whole mitochondrial genome sequences. By comparing the topologies of nuclear gene trees built using all gene copies and the mitochondrial tree, we were able to better resolve the phylogenetic relationships within the family Botiidae.

To our knowledge, no previous studies have estimated a timetree specifically focusing on the family Botiidae. A timetree is needed to know the divergence times of Botiidae, its subfamilies, genera, and species. It is also important if we want to investigate when the whole genome duplication (polyploidization) event might have happened. Moreover, botiid species have a very interesting distributional pattern. The diploid subfamily Leptobotiinae is endemic to East Asia, while the tetraploid subfamily Botiinae is mainly distributed in Southeast Asia and South Asia [15]. Both subfamilies contain about the same number of valid species (33 vs. 32; see [9]). A timetree will allow us to explore the biogeographical history of botiid fishes and may shed light on the potential roles that polyploidization might have played during the diversification of these fishes.

In summary, the objectives of this study are threefold: (1) to investigate whether members of the subfamily Botiinae are autotetraploids or allotetraploids and to assess how many polyploidization events have occurred during the evolution of this subfamily; (2) to reconstruct the phylogenetic relationships among botiid fishes using DNA sequences from both mitochondrial genomes and phased nuclear genes; and (3) to build a timetree and use it to reconstruct the biogeographical history of botiid fishes.

## 2. Materials and Methods

### 2.1. Taxon Sampling, DNA Extraction, PCR, Library Preparation, and Sequencing

A total of 21 new samples representing both subfamilies and all eight genera of the family Botiidae were used for sequencing in this study. The detailed sample information can be found in Tables S1 and S2. The total genomic DNA of these samples was extracted using the E.Z.N.A. Tissue DNA Kit (Omega Bio-tek, Inc., Norcross, GA, USA) following the manufacturer’s instructions. The whole mitochondrial genome (mitogenome) sequences were obtained using a gene capture method followed by next-generation sequencing [26,27]. Briefly, an aliquot of the extracted total genomic DNA (0.5–3 μg) of each sample was sheared to ~500 bp using a Covaris M220 Focused-ultrasonicator (Covaris, Inc., Woburn, MA, USA). After that, the dual-indexed genomic libraries were prepared, and they were subjected to one round of gene capture using RNA baits designed for vertebrate mitogenome enrichment [27].

For 14 out of the 21 samples, we tried to separate the different gene copies of five single-copy nuclear genes (i.e., EGR2B, EGR3, RAG1, RAG2, and IRBP2) using the “Next-Generation Sequencing followed by data phasing” method detailed in [28]. Primers and protocols used for the PCR amplification of these genes can be found in [29,30]. For each sample, PCR amplicons of the five nuclear genes were pooled together based on the brightness of bands on the agarose gel and then sheared to c.500 bp using acoustic ultrasonication on a Covaris M220 Focused-ultrasonicator (Covaris, Inc., Woburn, MA, USA). Dual-indexed Illumina sequencing libraries were then prepared for all samples following [26]. For the indexing step, the number of PCR cycles was set to five to minimize PCR-induced errors. The individual mitogenome libraries and nuclear gene libraries were pooled in equimolar ratios, respectively, and sent to the Interdisciplinary Center for Biotechnology Research of the University of Florida (UF-ICBR) for quality control and paired-end 300 bp sequencing on an Illumina MiSeq benchtop sequencer (Illumina, Inc., San Diego, CA, USA).

### 2.2. Mitogenome and Nuclear Gene Assembling

Adaptors and low-quality reads were trimmed from the demultiplexed raw reads using Trim Galore v0.6.4 [31]. For each sample, the duplicated reads were discarded, and unique reads were mapped to a reference in Geneious v.11.1.5 (https://www.geneious.com). For the mitogenome assembling of each species, the mitogenome sequence of a closely related species from the GenBank (*Ambastaia sidthimunki*, KP319024; *Botia lohachata*, KP729183; *Chromobotia macracanthus*, AB242163; *Sinibotia robusta*, KP979711; *Syncrossus beauforti*, AP011231; *Yasuhikotakia modesta*, KY131962; *Leptobotia elongata*, JQ230103; or *Parabotia fasciata*, KM393223) was used as the reference. For nuclear gene assembling, the following sequences from the GenBank were combined into a single FASTA file and used as the reference: EU409737 (*Botia dario*) for EGR2B, KP694684 (*Syncrossus beauforti*) for EGR3, EU711110 (*Sinibotia superciliaris*) for RAG1, KP696287 (*Yasuhikotakia lecontei*) for RAG2, and JN177266 (*Chromobotia macracanthus*) for IRBP2. For each sample, the mapping results of each gene were sorted out automatically by Geneious. We then deleted the references and reads that were not directly mapped to the reference for each read alignment. Next, we used the R package *copyseparator* 1.2.0 [28] to separate and assemble the two gene copies (if they existed) from the read alignment of each gene of each sample. The function “sep_assem” was used in most cases. The “copy_number” was set as 2, “read_length” was set as 300, and “overlap” was set as 225. In some cases, the function “copy_separate” was used, followed by manual assembly.

### 2.3. Datasets and Phylogenetic Analyses

#### 2.3.1. Mitogenome Dataset

The “Mitogenome dataset” was built by combining the 21 mitogenome sequences newly obtained in this study and the 99 mitogenome sequences (14 for Botiidae, 85 for outgroups) downloaded from the GenBank (Table 1 and Table S1). The 120 whole mitogenome sequences were aligned following the same procedures detailed in [32]. The final alignment is 14,888 bp in length, with 11,391 sites from the 13 protein-coding genes, 2096 sites from the two rRNA genes, and 1401 sites from the 22 tRNA genes.

#### 2.3.2. Individual Nuclear Gene Datasets

A separate dataset was built for each of the five nuclear genes. Taxon sampling information and characteristics of each dataset can be found in Table 1 and Table S2. For the genes EGR2B and EGR3, most tetraploid species have sequences from two gene copies (I and II). For the genes RAG1, RAG2, and IRBP2, only one gene copy was identified for each species.

#### 2.3.3. Concatenated Nuclear Gene Dataset

For each of the EGR2B dataset and the EGR3 dataset, a Copy I dataset was created by removing all Copy II sequences, and a Copy II dataset was created by removing all Copy I sequences. The “7-nuclear dataset” was then constructed by concatenating the EGR2B Copy I and II datasets, the EGR3 Copy I and II datasets, the RAG1 dataset, the RAG2 dataset, and the IRBP2 dataset. The 7-nuclear dataset contains 14 botiids and 18 outgroups and is 7240 bp in length (Table 1).

#### 2.3.4. All-Gene Dataset

The “All-gene dataset” was built by adding sequences from corresponding species in the Mitogenome dataset to the 7-nuclear dataset. The All-gene dataset contains 14 botiids and 18 outgroups and is 22,128 bp in length (Table 1).

#### 2.3.5. Phylogenetic Analyses

Maximum Likelihood (ML) analyses and bootstrap (MLBP) analyses were performed for each of the above datasets using RAxML v.8.0.26 [33,34,35]. For individual nuclear gene datasets, the sequence alignments were partitioned by codon positions, and the GTR + I + G substitution model was used. A total of 200 independent runs were performed. Based on preliminary results, we also built ML trees for RAG1, RAG2, and IRBP2 to show the placements of gene alleles of some species. The best partition schemes and partition models suggested by PartitionFinder v2.1.1 [36] were employed for the Mitogenome dataset and the two concatenated datasets. A total of 1000 independent runs were performed. The number of nonparametric bootstrap replications was set as 1000 for all MLBP analyses. The 50% majority rule consensus tree and bootstrap values (BP) were then calculated using PAUP *4.0.b10 [37].

### 2.4. Whole Genome Sequencing and Genome Profiling

Whole genome sequencing (WGS) and genome profiling were performed for two tetraploid species: *Chromobotia macracanthus* and *Yasuhikotakia modesta*. The WGS libraries were prepared using the NEBNext^®®^ Ultra™ II FS DNA Library Prep Kit (#E7805S; New England Biolabs, Ipswich, MA, USA). The dual-indexed libraries were then sent to UF-ICBR for quality control and paired-end 150 bp sequencing on an Illumina NovaSeq X Plus sequencer (Illumina, Inc., San Diego, CA, USA). Adaptors and low-quality reads were trimmed using Trim Galore v0.6.4 [31]. For each species, k-mers (length = 21) were counted from the short read data, and a histogram was exported using *Jellyfish* v.2.3.0 [38]. The histogram was then loaded into the online server of GenomeScope 2.0 (http://genomescope.org/genomescope2.0/; accessed on 1 March 2025; see [23]) to generate the GenomeScope profile plot. The ploidy level was set as 4 for both species. The above analyses were also performed for four other samples (i.e., *Yasuhikotakia modesta*, *Ambastaia sidthimunki*, *Botia almorhae*, and *B. kubotai*) based on the short read data (NCBI BioProject accession #: PRJNA1067307) obtained in [17].

### 2.5. Timetree Analyses

There is no reliable fossil from Botiidae to be used for the timetree calibration. To build a timetree for the family Botiidae, we must add taxa from other major families and build a timetree for the order Cypriniformes. Fortunately, some previous studies (e.g., [39,40,41]) have already performed such timetree analyses. Most of these studies (e.g., [39,40]) only used one or two botiid species to represent the family. Hirt et al. (2017), however, used ten botiid species in their timetree analyses for Cypriniformes [41]. Moreover, they collected sequence data from six nuclear genes and 154 species and used eight carefully selected fossils. In the current study, we decided to use the divergence time estimate of [41] for the most recent common ancestor (MRCA) of Botiidae (~51 Mya—million years ago) to infer the divergence times of all the botiid taxa we analyzed. We first generated 1000 random numbers following a normal distribution (mean = 51.0, sd = 6.08, so that 5th percentile = 41.0 and 95th percentile = 61.0) in R 4.4.2 [42], using the function rnorm, and then randomly picked 500 numbers that were larger than 41 but smaller than 61. Next, a total of 500 separate timetree analyses were performed in TreePL [43] using 41.0 Mya as the minimum age and each of the 500 numbers generated above as the maximum age for the MRCA of Botiidae. We pruned all non-Botiidae taxa from the best ML tree inferred from the Mitogenome dataset and used it as the input tree. The trees built based on the nuclear datasets and concatenated datasets were not used because they contain fewer botiid species. TreePL uses a penalized likelihood approach to generate timetrees in a Maximum Likelihood framework. It uses tree topologies as input files. Whether the trees were inferred from mitochondrial gene sequences or nuclear gene sequences does not impact the results. For the analyses, we performed cross-validation analyses using the “cv” and “randomcv” commands and tested the performance of the available optimization routines using the “prime” command. We then used the “thorough” command to ensure the preferred optimization routine converged. The 500 timetrees obtained were merged and imported into TreeAnnotator 2.7.7 [44] to generate the maximum clade credibility (MCC) tree (no burn-in; posterior probability limit, 0.0; median node heights).

### 2.6. Ancestral Range Reconstruction

Ancestral range reconstruction of the Botiidae was conducted in R 4.4.2 [42] using the package BioGeoBEARS [45]. The timetree built above was used in the analysis. Based on the current distribution range of botiid species, four areas were defined, i.e., A: Mainland Southeast Asia minus Myanmar (Thailand, Laos, Vietnam, etc.); B: Maritime Southeast Asia (Borneo, Sumatra, Java, etc.); C: South Asia (India, Bangladesh, Pakistan, etc.) plus Myanmar; and D: East Asia (mainly China). Because each area is large, the maximum number of ancestral areas allowed at each node was set as two. Distances among neighboring areas were measured in Google Earth Pro version 9.3.6. using the shortest distance between the assumed centers of distribution. The distance matrix was re-scaled by dividing all the distances by the shortest distance in the matrix. Values in the matrix were then rounded to the first decimal digit. This re-scaling and rounding can greatly reduce the impact of measurement uncertainty. A total of six models (DEC, DEC + J, DIVALIKE, DIVALIKE + J, BAYAREALIKE, and BAYAREALIKE + J) implemented in BioGeoBEARS were tested.

## 3. Results

### 3.1. Nuclear Gene Copies

We have identified two gene copies (Copies I and II) for the genes EGR2B and EGR3 for most species, while we only found one gene copy for the other three genes (i.e., RAG1, RAG2, and IRBP2). No indels were found in the EGR3 and IRBP2 gene alignments. In the RAG1 alignment, *Sinibotia pulchra* has an insertion at position 633. This insertion interrupts the reading frame and was ignored during phylogenetic analyses. In the RAG2 alignment, *Yasuhikotakia eos* has deletions at positions 956–958. In the EGR2B alignment, *Yasuhikotakia modesta* (Copy I) and *Y. eos* (Copy I) share the insertion from position 190 to position 192. *Yasuhikotakia modesta* (Copy I) has deletions at the following alignment positions: 223–240 and 472–489. *Botia lohachata* (Copy I) has deletions at positions 373–381. *Sinibotia robusta* (Copy I) and *S. pulchra* (Copy I) share deletions at positions 511–513. *Yasuhikotakia modesta* (Copy II) has deletions at positions 502–513.

### 3.2. Phylogenetic Relationships

**Figure 1:**
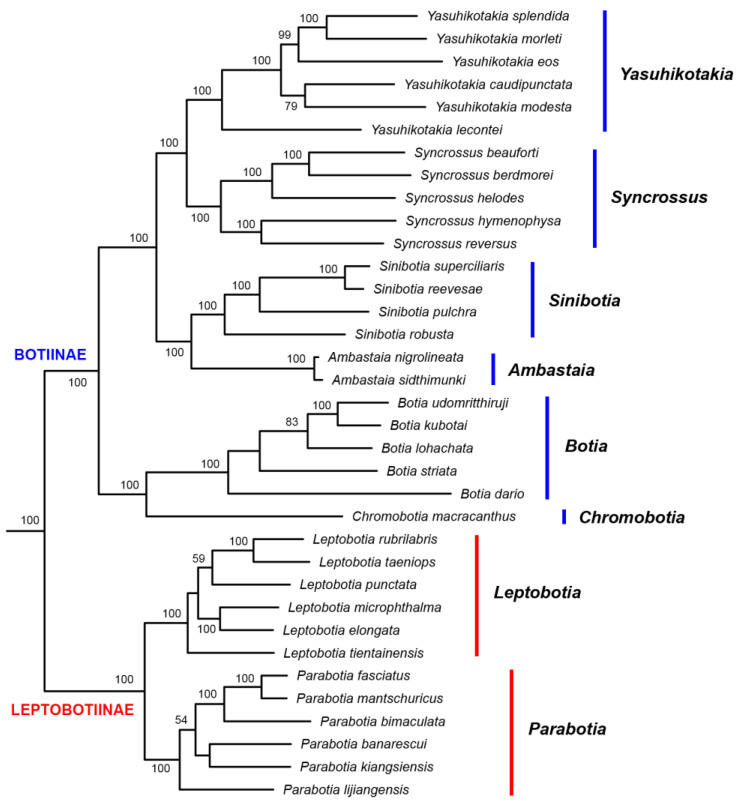
The best Maximum Likelihood tree (-*Ln*L = 458,271.170016) built based on the Mitogenome dataset. Only botiid species are shown. Numbers beside nodes are bootstrap support values (BP). Only those values ≥ 50% are shown.

**Figure 2:**
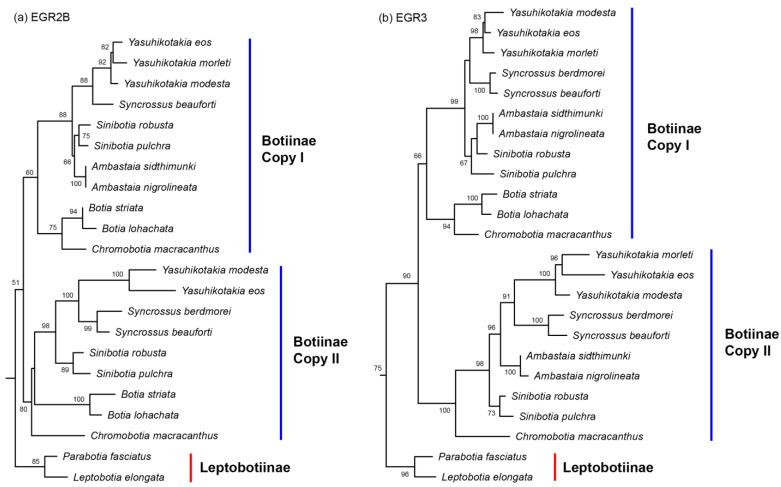
The best Maximum Likelihood trees built based on: (**a**) EGR2B dataset (-*Ln*L = 5871.561986), and (**b**) EGR3 dataset (-*Ln*L = 6741.165360). Only botiid species are shown. Numbers beside nodes are bootstrap support values (BP). Only those values ≥ 50% are shown.

**Figure 3:**
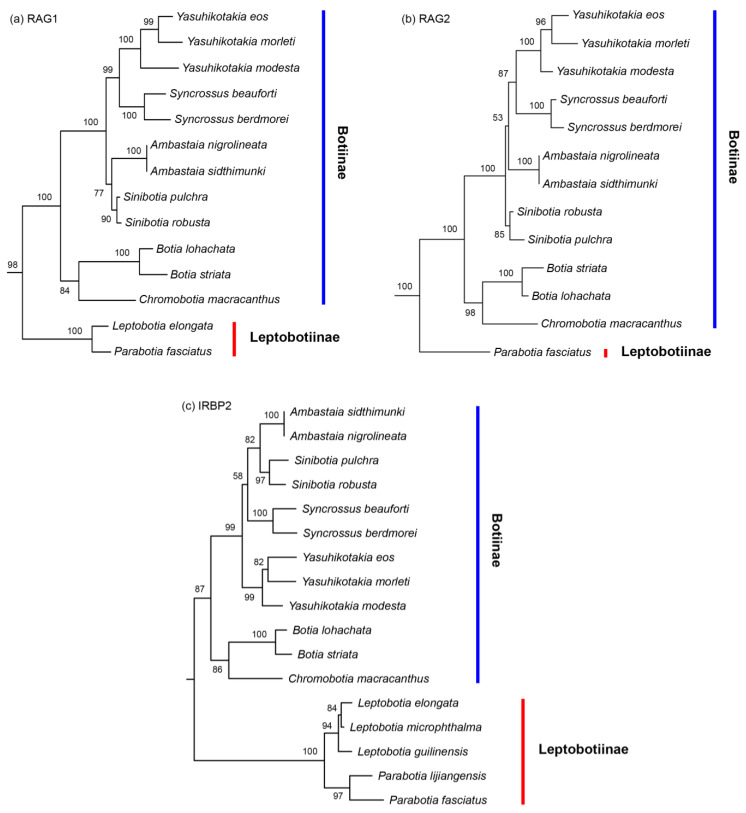
The best Maximum Likelihood trees built based on the (**a**) RAG1 dataset (-*Ln*L = 11,118.905157), (**b**) RAG2 dataset (-*Ln*L = 7967.061854), and (**c**) IRBP2 dataset (-*Ln*L = 6904.434141). Only botiid species are shown. Numbers beside nodes are bootstrap support values (BP). Only values ≥ 50% are shown. See Figures S1–S3 to see the trees with alleles of some species shown.

**Figure 4:**
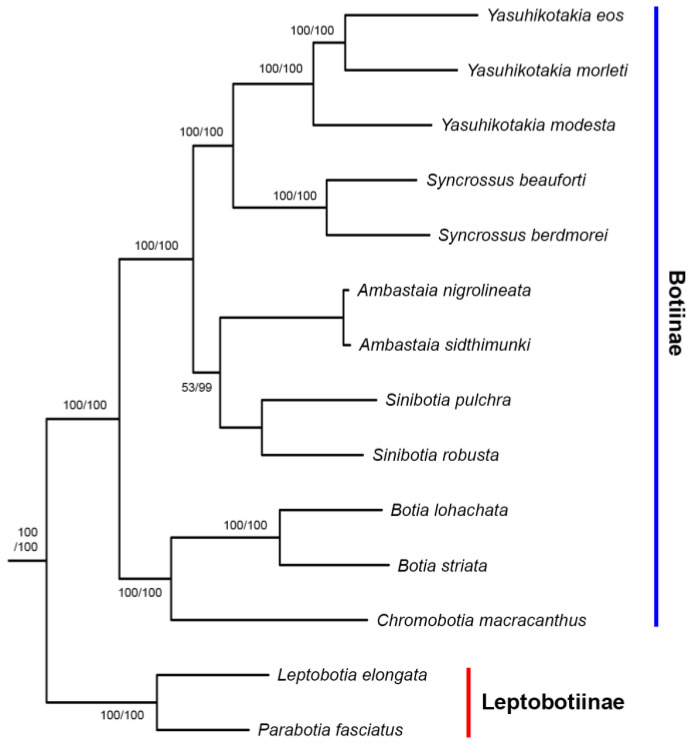
The best Maximum Likelihood trees built based on the 7-nuclear dataset (-*Ln*L = 47,826.851238) and the All-gene dataset (-*Ln*L = 196,328.028457). Only the latter tree is shown here because the two trees share the same topology. Only botiid species are shown. Numbers beside nodes are bootstrap support values (BP) for the 7-nuclear dataset (before slash) and the All-gene dataset (after slash). Only those values ≥ 50% are shown.

In the tree built from the Mitogenome dataset, the subfamilies Leptobotiinae and Botiinae formed two reciprocally monophyletic groups (Figure 1). Within Leptobotiinae, the two genera *Leptobotia* and *Parabotia* are also reciprocally monophyletic. Within Botiinae, besides the monotypic *Chromobotia*, the other five genera are all monophyletic. The sister group formed by *Ambastaia* and *Sinibotia* is sister to the sister group formed by *Syncrossus* and *Yasuhikotakia*. The group formed by these four genera is then sister to the sister group formed by *Chromobotia* and *Botia*. All but four nodes in the Botiidae are robustly supported (BP =99% or 100%). The generic-level relationships shown by the mitogenome tree also held true for the trees inferred from the 7-nuclear dataset, the All-gene dataset, the EGR2B dataset (Copy I), and the RAG1 dataset (Figure 2, Figure 3 and Figure 4). *Ambastaia* is not placed as the sister to *Sinibotia* in the RAG2 tree, *Yasuhikotakia* is not sister to *Syncrossus* in the IRBP2 tree, and *Chromobotia* is not sister to *Botia* in the EGR2B tree (Copy II). However, the relevant nodes have low bootstrap values (all <60%; see Figure 2 and Figure 3). In the EGR3 tree (Copy I), the two *Sinibotia* species did not form a monophyletic group. However, the relevant node is not strongly supported (67%; see Figure 2b). In the EGR3 tree (Copy II), *Ambastaia* and *Sinibotia* are not reciprocal sister groups, and this relationship is strongly supported (BP = 96%; see Figure 2b). The ML trees built for RAG1, RAG2, and IRBP2 with gene alleles shown can be found in Figures S1–S3.

### 3.3. Whole Genome Sequencing and GenomeScope Profile

**Figure 5:**
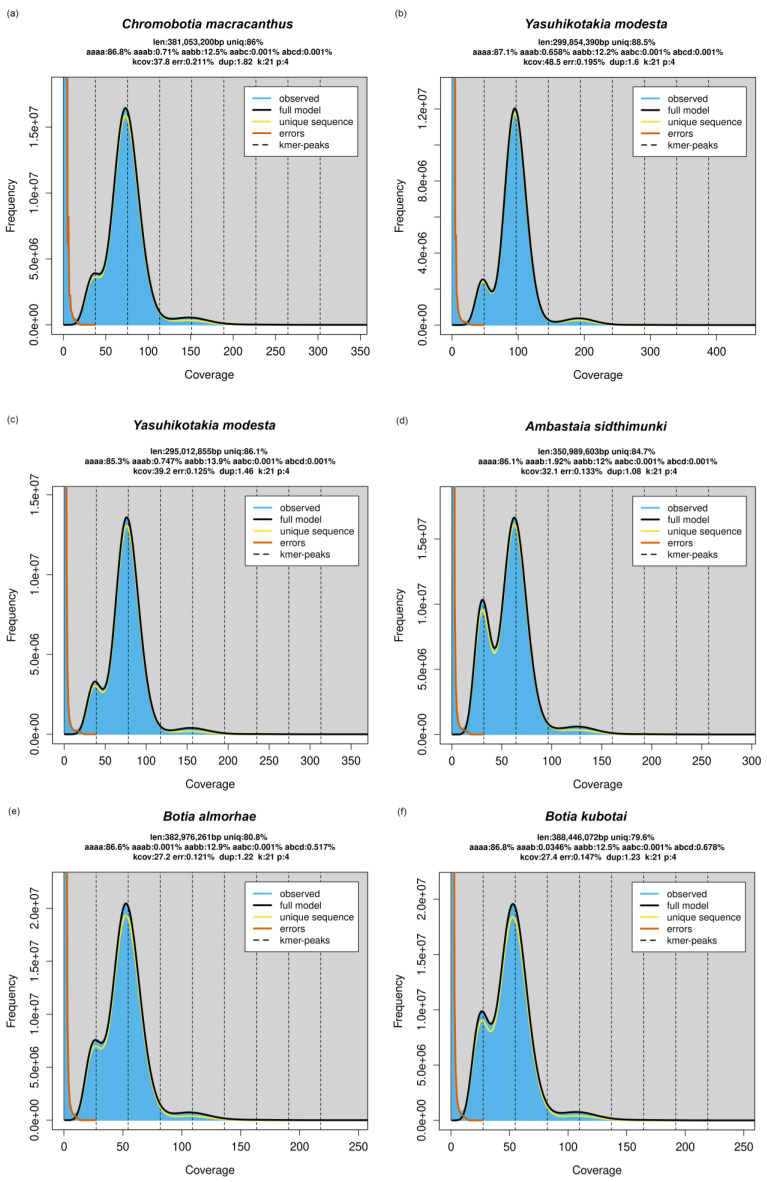
GenomeScope 2.0 profile plots for botiid fishes. (**a**,**b**) were based on data (NCBI BioProject accession #: PRJNA1257825) generated in the current study. (**c**–**f**) were based on data (NCBI BioProject accession #: PRJNA1067307) obtained by Bitsikas et al. (2024) [17]. For (**b**), the “average k-mer coverage” was set as 50 based on preliminary results. For other samples, this parameter was set as default (−1).

A total of 301,552,512 and 295,865,186 sequence reads have been produced by NovaSeq for *Chromobotia macracanthus* and *Yasuhikotakia modesta*, respectively. The GenomeScope profile plots for both species can be found in Figure 5a,b. For *C. macracanthus*, the estimated percentage for the heterozygosity form *aabb* is 12.5%, much higher than that of *aaab* (0.71%). For *Y. modesta*, the percentage of *aabb* (12.2%) is also much higher than that of *aaab* (0.658%). For the four samples from [17], the same pattern has been observed (Figure 5c–f).

### 3.4. Divergence Time Estimation

According to our timetree (Figure S4), the subfamily Botiinae began to diverge at 41.6 Mya, while the first split in the subfamily Leptobotiinae happened at 25.1 Mya. The MRCAs of the tetraploid genera *Botia*, *Sinibotia*, *Syncrossus*, and *Yasuhikotakia* all have similar ages; they are 22.5 Mya, 21.1 Mya, 21.5 Mya, and 21.8 Mya, respectively. The monotypic genus *Chromobotia* originated at 35.7 Mya. The two species of *Ambastaia* did not form until very recently (0.8 Mya). The two diploid genera *Parabotia* and *Leptobotia* began to diverge at 18.2 Mya and 15.6 Mya, respectively. The 95% Highest Posterior Density (HPD) intervals of those ages can be found in Figure S4.

### 3.5. Ancestral Range Reconstruction Results

**Figure 6:**
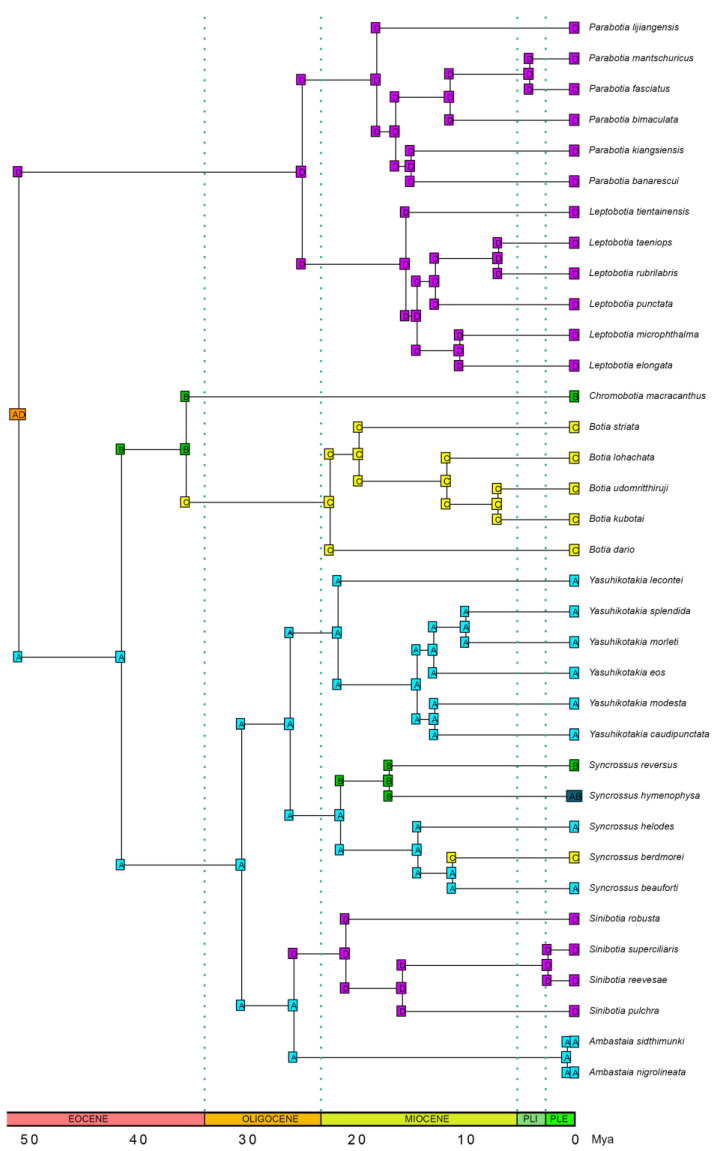
Biogeographical history of the family Botiidae inferred using BioGeoBEARS based on the DIVALIKE + J model. A: Southeast Asia (mainland minus Myanmar); B: Southeast Asia (Maritime); C: South Asia (plus Myanmar); D: East Asia.

Our ancestral range reconstruction results show that the analysis based on the model DIVALIKE + J returned the highest likelihood value (*Ln*L= −25.97). Botiidae might have an ancestral distribution in East Asia and Mainland Southeast Asia (Figure 6). After the split of Botiinae and Leptobotiinae, the former stayed in East Asia and later diverged into the current generic and specific diversity, while the latter dispersed to Maritime Southeast Asia, South Asia, and East Asia and diverged into the current generic and specific diversity during the process.

## 4. Discussion

### 4.1. The Origin of Polyploidy in Botiinae

For the three nuclear genes RAG1, RAG2, and IRBP2, we obtained sequences corresponding to a single gene copy in all botiine species analyzed (Figure 3). This could be due to the loss of one gene copy or the possibility that the PCR primers we used were copy-specific. Another explanation could be recombination between gene copies—i.e., homoeologous exchanges [46]—which may homogenize the sequences and obscure the presence of multiple copies. As a result, these three genes are uninformative for determining whether the subfamily Botiinae is of allopolyploid or autopolyploid origin. In contrast, for the nuclear genes EGR2B and EGR3, we successfully recovered two distinct copies for each gene in Botiinae, confirming that species within this subfamily are indeed tetraploids (Figure 2). However, because each gene copy formed a distinct clade and the two clades were sisters to each other in the phylogenetic trees, we are still unable to determine whether botiine species are allopolyploids or autopolyploids. In some known allotetraploid lineages, such as the family Catostomidae, similar tree topologies have been observed for five nuclear genes [28]. In autotetraploid lineages, such as salmonid fishes, some genes that evolved under the so-called AORe (Ancestral Ohnologue Resolution) model can also exhibit similar tree topologies [47]. Under the AORe model, rediploidization occurs in the common polyploid ancestor, and the ohnologues (duplicated gene copies) begin to diverge prior to the subsequent speciation events that give rise to descendant lineages.

Robertson et al. (2017) also proposed another evolutionary model specific to autopolyploids: the LORe (Lineage-specific Ohnologue Resolution) model [47]. Under this model, speciation predates the sequence divergence of ohnologues, leading to lineage-specific divergence patterns. That is because, in some genes, the rediploidization process took too long. When it was completed, speciation events had already taken place [48,49,50]. Under the LORe model, both gene copy sequences of one or more (but not all) descendant species of a tetraploid group should be clustered together in the gene tree. Multiple such small clusters may appear in the tree. Within each cluster, if there is more than one sample, all Copy I sequences are grouped together, and all Copy II sequences are grouped together; if there is only one sample, then its Copy I and Copy II sequences are sisters to each other. In our current study, the two alleles of the RAG2 sequences of *Sinibotia robusta* and *Ambastaia sidthimunki* differ by 25 bp and 17 bp, respectively. For each species, its two alleles were sisters to each other in the phylogenetic tree (Figure S2). Should the two alleles of these species be treated as two distinct gene copies? We do not know, as there is no clear boundary between alleles and gene copies. If treated as separate gene copies, the RAG2 gene in these species may have evolved under the LORe model, which would be a sign of autopolyploidy. However, we did not identify any larger clades in our trees that would indicate widespread delayed rediploidization. In summary, based on the DNA sequences of the five single-copy nuclear genes analyzed and the resulting gene trees, we are unable to draw a definitive conclusion as to whether the subfamily Botiinae is of allopolyploid or autopolyploid origin. Only five nuclear loci were used in this study. Future research employing hundreds of nuclear loci may provide a clearer resolution of this issue from a phylogenomic perspective.

The GenomeScope profile plots for five botiine species—*Chromobotia macracanthus*, *Yasuhikotakia modesta*, *Ambastaia sidthimunki*, *Botia almorhae*, and *B. kubotai*—representing four of six genera of the subfamily Botiinae, revealed that the estimated percentage of the heterozygosity form *aabb* is consistently much higher than that of *aaab* (Figure 5). According to [23], such a pattern is indicative of an allopolyploid origin. That is because, in allotetraploids, the two subgenomes are from different parental species. During meiosis, homologous chromosomes from the same subgenome tend to pair preferentially, often resulting in the observation of two homologs from each subgenome after recombination. In contrast, autotetraploids possess subgenomes originating from the same parental species. During meiosis, chromosomes from both subgenomes may engage in polysomic inheritance, frequently forming quadrivalents. This pattern reduces the likelihood of recovering exactly two homologs from each subgenome following recombination. A more detailed explanation can be found in [23]. Based on this genomic evidence, it appears that fishes in the subfamily Botiinae likely had an allotetraploid origin. Nevertheless, we acknowledge that drawing definitive conclusions about the nature of polyploidy can be challenging. Features once thought to be exclusive to autopolyploids—such as polysomic inheritance and multivalent formation—have also been reported in some allopolyploids [20,51].

Because the two gene copies are sisters to each other in both the EGR2B tree and the EGR3 tree (Figure 2), it is not possible to distinguish which copy is maternally inherited and which is paternally inherited. Consequently, we are unable to identify the potential maternal or paternal progenitor of the subfamily Botiinae. It is likely that one of the progenitor lineages is now extinct. Furthermore, the subfamily Leptobotiinae is unlikely to represent either the maternal or the paternal progenitor of Botiinae, as it did not group with either of the Botiinae gene copies in the EGR2B or EGR3 trees.

Allopolyploidy appears to be the predominant mechanism underlying the origin of polyploidy in fishes. To date, only a few fish groups have been confirmed to be autopolyploids, including Salmonidae, Schizothoracinae, Schizopygopsinae, and some species of Acipenseriformes [8,47,52,53,54]. Besides Botiinae, we already know that all the following polyploid fish groups are allopolyploids: Probarbinae, Cyprininae, Spinibarbinae, tetraploid Torinae, hexaploid Torinae, tetraploid Barbinae, hexaploid Barbinae, and Catostomidae [7,8,28,55]. Some sturgeons (Acipenseriformes) may also be of allopolyploid origin [56]. Our preliminary data also suggest that the tetraploid Smiliogasterinae are allopolyploids as well. In plants, there is still debate on whether allopolyploids are also more common than autopolyploids [20,51,57,58].

The topologies of the EGR2B and EGR3 gene trees clearly indicate that the tetraploidization of the subfamily Botiinae occurred only once, during the formation of the common ancestor of this subfamily (Figure 2). Šlechtová et al. (2006) expressed the same opinion after mapping ploidy levels on their mitochondrial phylogeny, without considering that the same pattern could also be produced if multiple independent tetraploidization events occurred within Botiinae [15]. Sember et al. (2018) reached the same conclusion as ours by analyzing the dynamics of some tandemly repeated DNA sequences and detected a high degree of rediploidization [16]. Based on our timetree, this tetraploidization event might have happened after the Botiinae originated 51.0 Mya and before it began to diversify 41.6 Mya, corresponding to the early to middle Eocene (Figure S4). Therefore, the polyploidization of Botiinae appears to predate most, if not all, polyploidization events in Cyprinidae (<44 Mya; see [8]) but is later than the polyploidization of Catostomidae (>63 Mya; see [59]). Our biogeographical reconstruction suggests that this polyploidization event likely took place in Mainland Southeast Asia, which is home to several large river systems (e.g., Mekong River and Chao Phraya River) (Figure 6). Interestingly, the polyploidization of the small allotetraploid cyprinid subfamily Probarbinae might have also occurred in Mainland Southeast Asia, as all its extant species are restricted to this region [9].

### 4.2. Phylogenetic Relationships

Previous phylogenetic studies on Botiidae that used mitochondrial genes (e.g., [15,16,24,25]) usually only used one or a few genes (e.g., Cytochrome *b*, 12S rRNA gene). In the current study, however, the whole mitochondrial genome sequences were employed. All generic-level relationships were resolved with 100% bootstrap support, and most other ingroup nodes were also robustly supported (Figure 1). This is not the case for any previous phylogenetic studies on Botiidae. Moreover, we also tried to separate the gene copies for five nuclear genes and built phylogenetic trees based on their sequences. This has not been achieved by any previous studies that used nuclear genes (e.g., [16,25]), which makes the results from these studies potentially plagued with paralog issues.

All phylogenetic trees built in this study (Figure 1, Figure 2 and Figure 3), except for the EGR3 tree (Copy I), strongly supported or at least did not reject the following relationships among Botiidae. Leptobotiinae/Botiinae, *Leptobotia*/*Parabotia*, *Chromobotia*/*Botia*, *Yasuhikotakia*/*Syncrossus*, and *Sinibotia*/*Ambastaia* are all sister taxa. Clades formed by the last two generic pairs are sisters to each other. The clade formed by these four genera is sister to the clade formed by *Chromobotia*/*Botia*. The above relationships have also been shown by the RAG1 tree of Bohlen et al. (2016) [25]. In the EGR3 tree (Copy I), *Ambastaia* and *Sinibotia* did not form a sister group (Figure 3). That might be the result of incomplete lineage sorting.

All our phylogenetic trees indicated that the two species of *Ambastaia*, *A. sidthimunki* and *A. sidthimunki*, are very closely related to each other. Especially in the trees built based on the RAG1 dataset, the RAG2 dataset, and the IRBP2 dataset, the two alleles of both species were mixed with each other (Figures S1–S3), which is a sign that they have diverged from their common ancestor relatively recently. This is supported by our timetree, which shows that the two species diverged only 0.8 million years ago (Figure S4).

Due to resource limitations, this study only included 35 species in the mitogenome dataset and 14 species in the nuclear datasets, representing 53.8% and 21.5% of all currently recognized botiid species, respectively. Furthermore, only five nuclear genes were analyzed. Future studies incorporating broader taxon sampling and more extensive genomic data will be essential for a more comprehensive understanding of phylogenetic relationships within the family Botiidae.

### 4.3. Biogeographical History

According to our ancestral range reconstruction results (Figure 6), the family Botiidae likely originated in East Asia and Mainland Southeast Asia around 51 Mya. This time frame coincides with the initial collision of the Indian plate with the Eurasian plate [60]. The ancestors of Indian botiid fishes likely dispersed from Southeast Asia in two distinct waves. The first wave may have occurred during the Eocene, introducing the common ancestor of *Botia* to the Indian subcontinent. The second wave likely took place in the middle Miocene, bringing *Syncrossus* into the region. Most Chinese botiid species are diploids belonging to the subfamily Leptobotiinae, which originated during the initial divergence of Botiidae. In contrast, the common ancestor of the tetraploid genus *Sinibotia* likely dispersed from Mainland Southeast Asia during the Early Miocene. The presence of *Chromobotia* and *Syncrossus* on the large islands of Southeast Asia likely resulted from two separate dispersal events from the mainland. The first wave might have occurred in the late Eocene to early Oligocene, while the second wave likely took place during the Miocene.

The DIVALIKE + J model inferred a long-distance dispersal event from Maritime Southeast Asia (B) to South Asia and Myanmar (C) along the branch leading to *Chromobotia* and *Botia* (Figure 6). This inference is based on the sister relationship between *Chromobotia*—which is currently distributed in Sumatra and Borneo—and *Botia*, which occurs in South Asia and Myanmar. This dispersal likely took place during the late Eocene (Figure 6). Although the model did not explicitly reconstruct the route, we propose that the dispersal may have occurred via the nearby Malay Peninsula, which could have served as a stepping stone. We agree with Šlechtová et al. (2006) that *Chromobotia* and *Botia* might once have had a broader and continuous distribution [17].

The family Botiidae began to diverge around 51.0 Mya. Sometime after this, the ancestor of the subfamily Botiinae became tetraploid through hybridization followed by whole genome duplication. Subsequently, around 41.6 Mya, the subfamily Botiinae began to diverge. These major events occurred during the early to middle Eocene. This period was part of a global greenhouse phase, characterized by elevated atmospheric CO₂ levels and high global temperatures [61]. At the same time, the region was undergoing significant tectonic shifts, driven by the collision of the Indo-Australian plate with the Eurasian plate [60,62]. The tectonic processes also led to the development of complex river systems and the establishment of new ecological niches, promoting the diversification of freshwater fish species. The reorganization of river systems might have created opportunities for the maternal progenitor and the paternal progenitor of the Botiinae to meet and hybridize with each other and eventually form the tetraploid ancestor of the subfamily.

The subfamily Leptobotiinae began to diverge around 25.1 Mya, during the late Oligocene. East Asia underwent significant geological and climatic transformations during this period, which might have contributed to the diversification of the subfamily. Globally, this period was marked by a cooler and drier climate [63]. In East Asia, the ongoing collision between the Indian and Eurasian plates resulted in the progressive uplift of the Tibetan Plateau, reshaping regional topography and altering drainage patterns [64,65]. These tectonic processes played an important role in the early development of the East Asian monsoon system and the formation of new river systems and freshwater habitats [66], which later might have contributed to the further diversification of the subfamily Leptobotiinae.

## 5. Conclusions

Previous studies have remained inconclusive about whether the tetraploid subfamily Botiinae originated through autopolyploidy or allopolyploidy. In the present study, we employed both a phylogeny-based genetic approach and a k-mer-based genomic method to address this question. Our results indicate that Botiinae likely have an allopolyploid origin, with the tetraploidization event occurring only once in the common ancestor of the subfamily. Phylogenetic trees inferred from mitochondrial genome sequences and five phased nuclear gene sequences were largely congruent, supporting the following sister group relationships: Leptobotiinae/Botiinae, *Leptobotia*/*Parabotia*, *Chromobotia*/*Botia*, *Yasuhikotakia*/*Syncrossus*, and *Sinibotia*/*Ambastaia*. We also estimated divergence times and reconstructed the ancestral range distribution of botiid fishes. Our analyses suggest that the family Botiidae likely originated in East Asia and Mainland Southeast Asia approximately 51 million years ago, followed by its dispersal into South Asia and the islands of Southeast Asia, ultimately giving rise to the group’s current generic and species diversity. Future studies will need to expand taxon sampling and incorporate data from more nuclear loci. This is critical for improving our understanding of the phylogenetic relationships, polyploid evolution, and biogeography of botiid fishes.

## Figures and Tables

**Table 1:** Taxon sampling and characteristics of different datasets used in this study.

	Mitogenome	EGR2B	EGR3	RAG1	RAG2	IRBP2	7-Nuclear	All-Gene
Botiidae	35	14	14	14	13	17	14	14
Outgroup	85	18	17	18	10	17	18	18
Total species	120	32	31	32	23	34	32	32
Total sequences	120	40	41	32	23	34	32	32
Nucleotides (bp)	14,888	828	953	1497	1314	864	7240	22,128
Variable characters (bp)	8372	348	393	650	613	446	3070	10,266
Parsimony-informative characters (bp)	7188	246	288	513	396	320	2155	7909

## Data Availability

All mitochondrial and nuclear gene sequence data used in this study can be found in GenBank (accession numbers are provided in Tables S1 and S2). The short Illumina read data used in this study can be found in NCBI’s Sequence Read Archive (SRA) with the BioProject accession #: PRJNA1257825 and PRJNA1067307.

## Supplementary Materials

The following supporting information can be downloaded at https://www.mdpi.com/article/10.3390/biology14050531/s1: Figure S1: Maximum Likelihood tree (-*Ln*L = 11,796.194434) built based on the nuclear RAG1 dataset; Figure S2: Maximum Likelihood tree (-*Ln*L = 8541.995023) built based on the nuclear RAG2 dataset; Figure S3: Maximum Likelihood tree (-*Ln*L = 6922.093239) built based on the nuclear IRBP2 dataset; Figure S4: Divergence time estimations for Botiidae; Table S1: Sample information for the mitogenome dataset; Table S2: Sample information for the nuclear dataset.
