# Supplementary material for: Origin of Polyploidy, Phylogenetic Relationships, and Biogeography of Botiid Fishes (Teleostei: Cypriniformes)
Source: Biology (Basel). 2025 May 11;14(5):531. doi: 10.3390/biology14050531 (PMC12109351; doi:10.3390/biology14050531)
Supplement: Supplementary file 1 [file biology-14-00531-s001.zip › Supplementary figure legends.pdf]

Supplementary figure legends

Fig. S1 Maximum Likelihood tree (-*Ln*L = 11796.194434) built based on the nuclear RAG1 dataset. Numbers beside nodes are bootstrap support values (BP). Only those values ≥ 50% are shown. The alleles of six species have multiple versions (v1-v4) depending on how they are assembled.

Fig. S2 Maximum Likelihood tree (-*Ln*L = 8541.995023) built based on the nuclear RAG2 dataset. Numbers beside nodes are bootstrap support values (BP). Only those values ≥ 50% are shown. The alleles of two species have multiple versions (v1-v2) depending on how they are assembled.

Fig. S3 Maximum Likelihood tree (-*Ln*L = 6922.093239) built based on the nuclear IRBP2 dataset. Numbers beside nodes are bootstrap support values (BP). Only those values ≥ 50% are shown. The alleles of two species have multiple versions (v1-v2) depending on how they are assembled.

Fig. S4 Divergence time estimations for Botiidae. Numbers at nodes denote divergence time estimates (unit: million years ago). Horizontal bars denote 95% Highest Posterior Density (HPD) intervals of node ages. PALEO: Paleogene; PLI: Pliocene; PLE: Pleistocene. Holocene not shown.
