# Supplementary figures and images for: Origin of Polyploidy, Phylogenetic Relationships, and Biogeography of Botiid Fishes (Teleostei: Cypriniformes)
Source: Biology (Basel). 2025 May 11;14(5):531. doi: 10.3390/biology14050531 (PMC12109351; doi:10.3390/biology14050531)

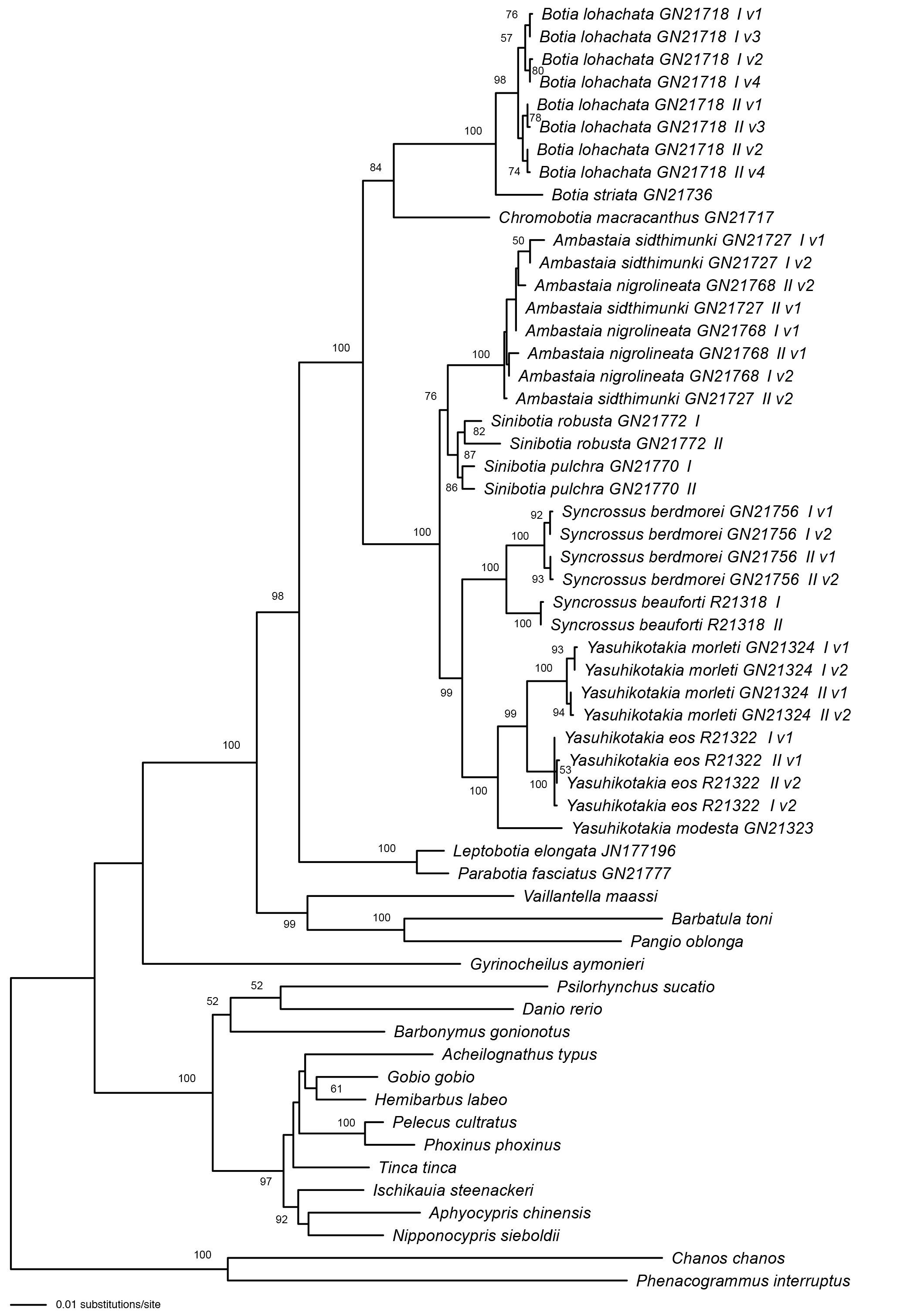

Supplement: Supplementary file 1 [file biology-14-00531-s001.zip › Fig. S1 Botiidae RAG1 ML tree show alleles.tif]

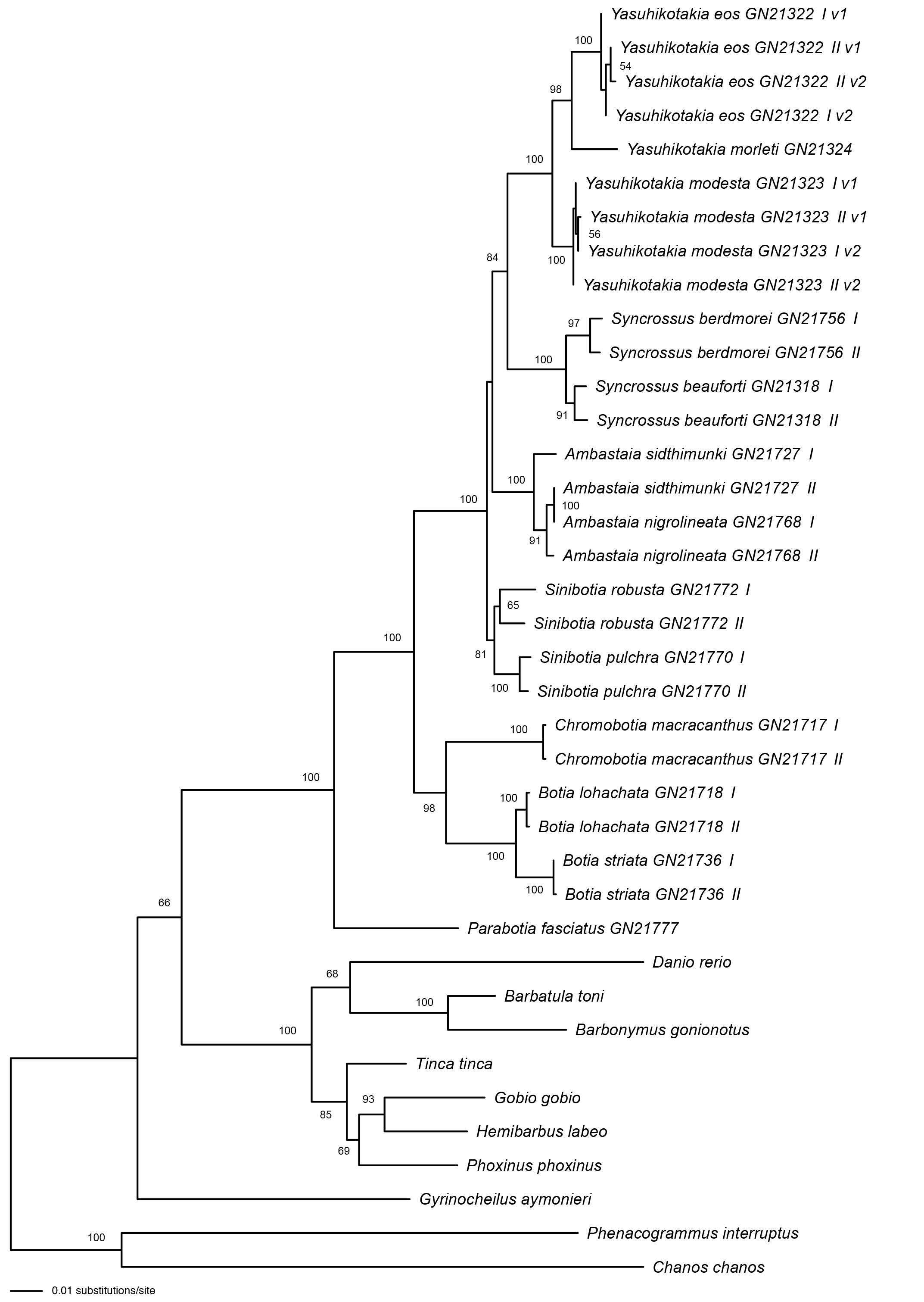

Supplement: Supplementary file 1 [file biology-14-00531-s001.zip › Fig. S2 Botiidae RAG2 ML tree show alleles.tif]

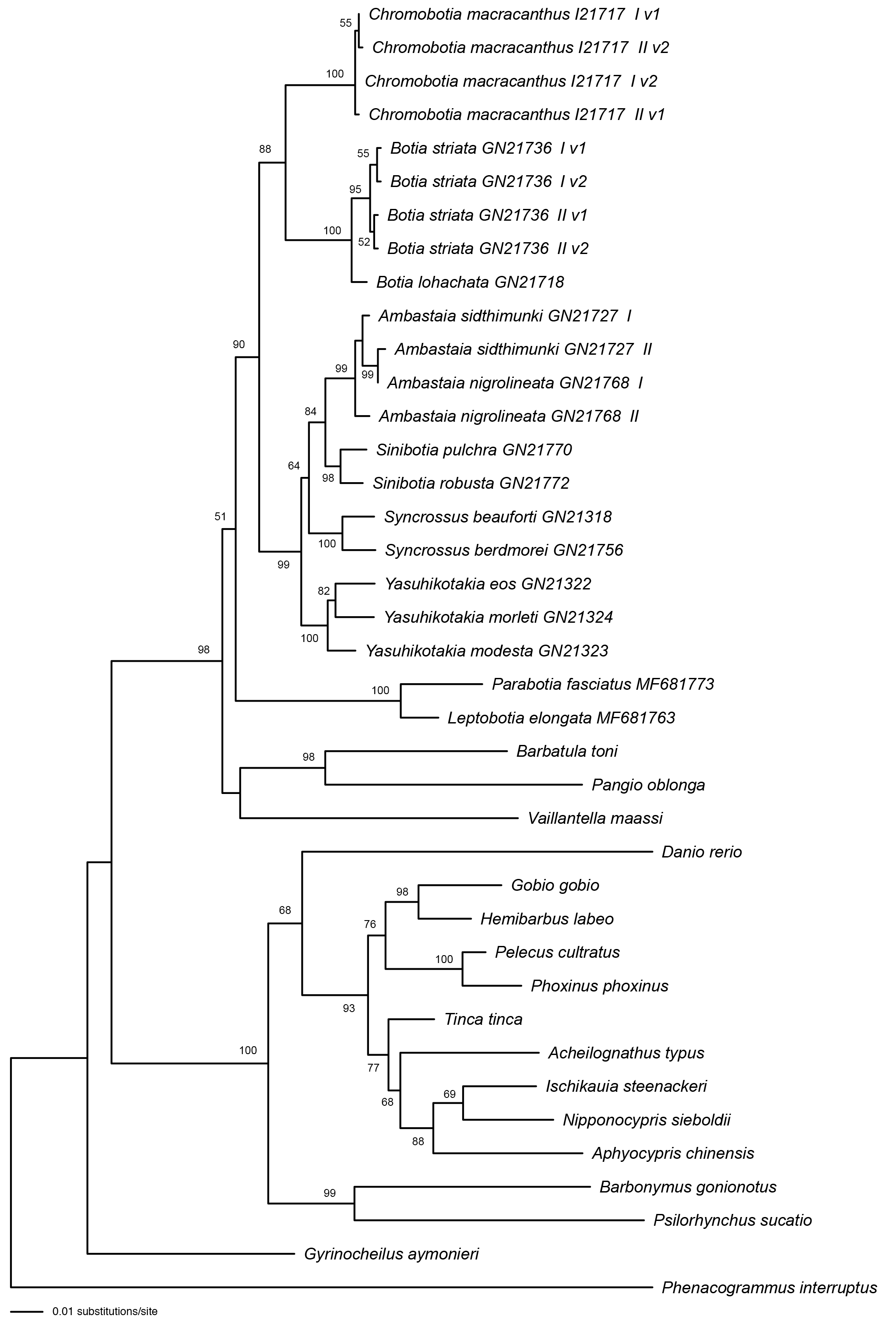

Supplement: Supplementary file 1 [file biology-14-00531-s001.zip › Fig. S3 Botiidae IRBP2 ML tree show alleles.tif]

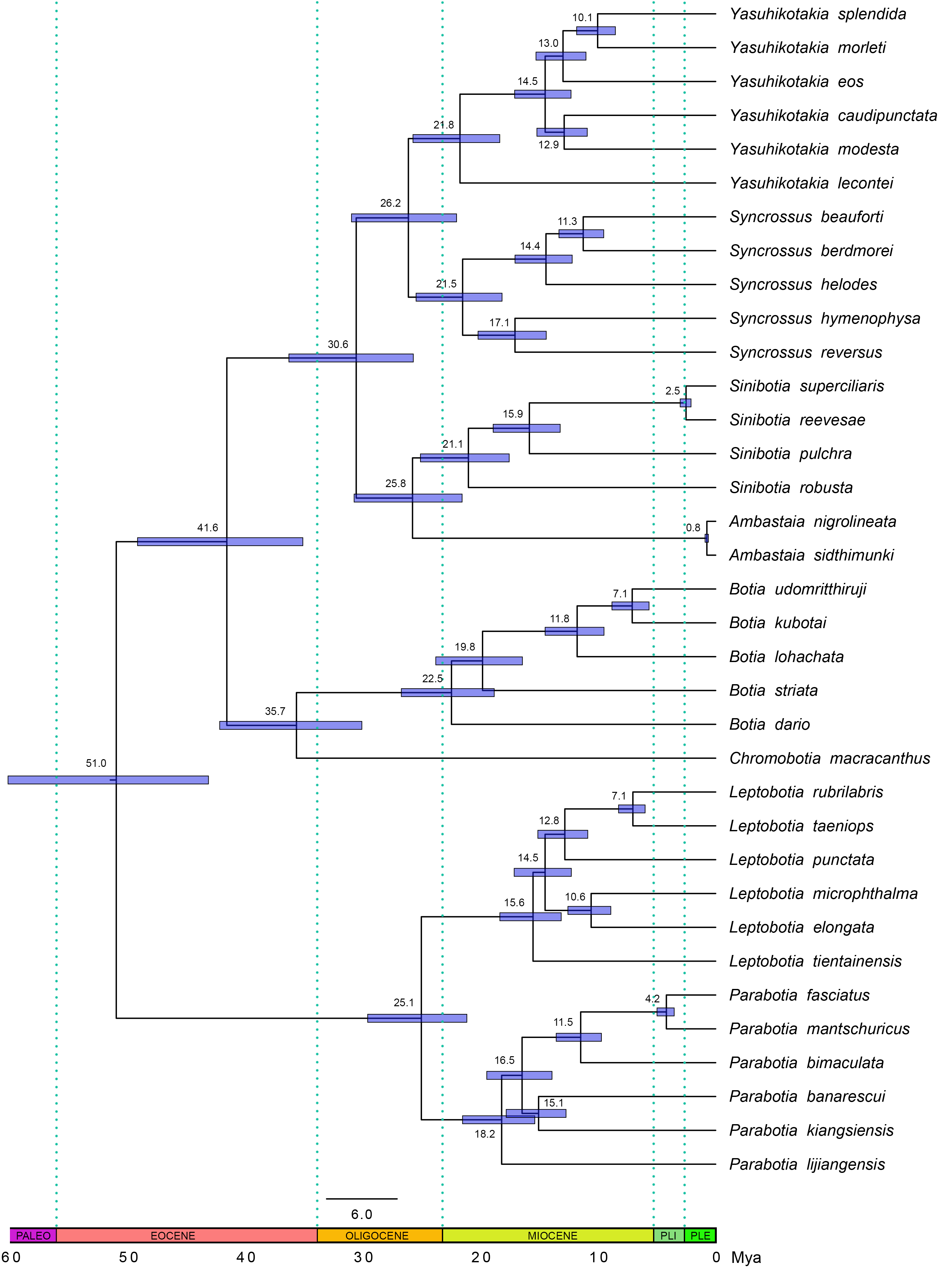

Supplement: Supplementary file 1 [file biology-14-00531-s001.zip › Fig. S4 Divergence time estimations for Botiidae.tif]
